# Urban health inequality in shifting environment: systematic review on the impact of gentrification on residents' health

**DOI:** 10.3389/fpubh.2023.1154515

**Published:** 2023-07-20

**Authors:** Sun Delong

**Affiliations:** ^1^Key Laboratory of Ecology and Energy Saving Study of Dense Habitat, Ministry of Education, Shanghai, China; ^2^School of Architecture, Tianjin University, Tianjin, China

**Keywords:** systematic review, gentrification, changing neighborhoods, health, inequality

## Abstract

The impacts of changing neighborhoods, and the influence of neighborhood stability on residents' health have not received enough attention in the literature; one of the most important aspects is gentrification. Research on the impact of gentrification on residents' health has gradually increased in recent years, mainly from North America. Based on the guidelines of PRISMA 2020 and SCIE, 66 papers were included for analysis, six aspects of selected studies are discussed: the research design, theoretical framework, methods of analysis, definition and measurement of gentrification effects, and impact pathways. In general, most of the literature in this field can be seen as using an ecological research design, of which cross-sectional research accounts for a large proportion. The identified effects vary in their direction as well as strength due to difference in population, temporal, and geographical characteristics. Gentrification could affect health outcomes through the combination of economic, social, and physical environment factors. Existing research could be improved in the following aspects: (1) The definition and measurement of gentrification should be both generic and site-specific; Various measurement methods should be compared to enhance the robustness of the results. Furthermore, more consideration should be given to the impact of spatial issues; (2) As for health outcomes, it is suggested to expand the scope of the discussion of health outcomes and strengthen the biological explanation of the influencing mechanisms. It is also necessary to determine the research time points according to the characteristics of the incubation period of different diseases; (3) As for research design, applying longitudinal research design is more likely to improve the reliability; (4) Theoretical frameworks should be addressed to link the definition and measurement of gentrification, patterns of health outcomes, methodology and pathways.

## 1. Introduction

Health disparities and inequalities due to differences in race and socioeconomic status have become important obstacles to the sustainable development of cities around the world. For instance, in the United States, mortality rates, chronic diseases, and severe mental illness among impoverished populations and ethnic minorities, largely represented by black people, remain constantly high, making them important determinants affecting health. The evaluation of individuals' health cannot be carried out without taking into account the imbalanced distribution of social opportunities and limitations that individuals are exposed to, as these factors exert a significant impact on health-related behaviors of individuals (1). Current research on health inequality covers sociology, epidemiology, geography, urban planning, among other fields. The neighborhood we live can act as a critical social determinant of health (1). Neighborhood environment, considered as one of the most important units of daily life for residents, and an important contributor to health differentiation, has gradually received attention from many fields. Revealing how urban policies and social processes affect residents' health through the mediation of the neighborhood environment, and then deriving strategies to promote health equity by improving this context, has become an important point of interest for scholars globally. In addition to research on the stable neighborhood environment, studies of the changing neighborhood environment are also important, and one of the most influential phenomena is gentrification.

### 1.1. Theoretical background

At the heart of gentrification is the reconstruction of urban social space (2–4) through reinvestment in previously declining areas, generating extensive environmental, social, and industrial upgrading, whether intentional or not. This process is often manifested by new housing projects, cultural facilities, and commercial venues that reflect the consumption, entertainment, and housing tastes of new residents (the “middle class,” often privileged residents with high-income and ethnic white), in which large-scale government-led or private investment community redevelopment can produce dramatic gentrification. Gentrification process evolves in three cycles of human mobility, public policy and investment, and private capital flow (5). This phenomenon has attracted the interest of urban policy makers with the initial intention in enabling environmental improvements through reinvestment rather than causing neighborhood instability, accelerating residential mobility thus putting population health at risk. This instability derives mainly from involuntary displacements induced by gentrification, however, the relationship between gentrification and displacements is a controversial issue and hence a key to clarifying the relationship between gentrification and health.

Gentrification-induced displacement may be a key determinant affecting the health of the population and involuntary displacement can force churning moves (6) of low-income tenants, and these often mean a series of downward moves, whereas the housing instability caused by such displacement also has a negative impact on both the move-out and the move-in neighborhoods (7). Although a review of the relationship between gentrification and displacement is beyond the scope of this paper, this association provides some theoretical background to the effects of gentrification on population health. Numerous studies have found no significant correlation between gentrification and high rates of mobility (8–14). The willingness to live with the increased cost of living due to gentrification while enjoying its benefits, such as desirable location and better employment opportunities, if they can afford it, may reduce rates of mobility (15). Low-income groups, on the other hand, will have relatively high rates of out-migration in both gentrifying and non-gentrifying neighborhoods due to instable housing and source of income (16). Meanwhile, it is also challenging to measure displacement, especially in cross-sectional data, for the migration of long-term residents tends to be a slow process of being replaced and not necessarily displaced (9, 17, 18). In addition, displacement can also occur before, during, or after gentrification (19, 20). As for new-build gentrification, which is more common in developing countries, displacement occurs before the sign of gentrification, and if gentrification occurs on brownfield or vacant lots, where the original land use is not residential, displacement is no longer applicable (21). Nevertheless, recent research on the patterns and quality of displacement has further revealed the more nuanced conclusion on inequalities caused by gentrification: disadvantaged migrants displaced from gentrifying neighborhoods are more likely to move into deprived neighborhoods, a phenomenon that is more common in advanced gentrifying neighborhoods (15).

Although displacement is not necessarily a consequence of gentrification, some studies have further revealed that displacement, in a broad sense, is not limited to housing, but also involves employment, access to amenities, and interpersonal relationships in the form of indirect displacement, such as displacement pressures, exclusionary displacement, socio-cultural displacement and commercial displacement (20). For instance, exclusionary displacement does not necessarily lead to migration, but it places a financial burden on households in gentrifying neighborhoods and may cause generational displacement, which can be viewed as another form of residential displacement through which the offspring of long-time residents cannot live in the same neighborhood as their parents due to the difficulty in paying the cost of living after financially independent (22). The demographic and industrial shifts that accompany gentrification can lead to a loss of sense of place and identity, resulting in socio-cultural displacement (22–24). As the decline in neighborhood livability (25), residential displacement may come along. Commercial displacement occurs when local amenities in gentrifying areas involuntarily move out due to high rents or loss of certain customer groups, which often goes hand in hand with residential displacement (26).

In addition, existing research reveals that structural determinant such as racial discrimination and mechanisms of racial stratification also play a non-negligible role (27), operating at both the individual and neighborhood levels. At the individual level, in projects such as brownfield redevelopment, blacks have a lower willingness to pay than whites and are more likely to be victims of displacement (28), thereby resulting in environmental injustice. At the neighborhood level, because racial stratification mechanisms shape neighborhood valuation and selection, financial disadvantaged residents from gentrifying neighborhoods with a higher prevalence of Black ethnicity have a disproportionately high probability of moving to similarly composed non-gentrifying neighborhoods or to even more disadvantaged neighborhoods (29), which constrains mobility choices (27).

In summary, direct displacement can generally be seen as a potential, but not ubiquitous outcome of gentrification, and indirect displacement is more frequently demonstrated as the crucial factor inducing inequality. Given that, several theoretical underpinnings of gentrification's exacerbation of health disparities can be derived from the existing literature:

First, at the economic level, higher cost of living, and the ensuing residential or exclusionary displacement increase the likelihood of moving downward for disadvantaged residents once they migrate, thus reducing their payments for essential wellness resources or depriving them of the access to healthy environment, which is consistent with social determination mechanism.

Second, at the social level, the socio-cultural displacement caused by gentrification generates demographic shifts that alter the social network and culture identity of the neighborhood, increasing health risk factors associated with psychological distress. This phenomenon can be explained by psychosocial theory framework. This framework integrates social and personal indicators, emphasizing the role of specific social relationship in altering the probability of people contracting diseases, shifting the focus from endogenous biological factors to the impact of interpersonal interactions (30, 31). Such studies highlight the impact of gentrification on health through its influence on cultural and social relations, and thus the sociocultural characteristics of neighborhoods can be used to counteract the negative effects of gentrification. Some of the important concepts include social capital and collective efficacy (32, 33), while mechanism such as social disorganization, which may result in criminal behavior, is important mediator of the health effects of gentrification (33–35).

Third, physical landscape change and gentrification are often mutual causal and effect, and the two could be manipulated as intentional urban strategy attracting new investment in progresses such as urban redevelopment. For instance, the resulting commercial displacement undermines access to essential services for long-term residents, cuts them off from their everyday needs and aggravates structural conflicts, thus compromising their wellness resources or health behaviors. This phenomenon can be explained by the social production of disease/political economy of health framework. The emphasis of these studies is on privileges shaped by top-down political-economic institutions and their decision-making, which is at the root of health inequalities, including structural barriers and conflicts that affect health (33, 36). The focus is on how social justice, class differences, and power relations between institutions and people determine health segregation. The more representative branch is the political ecology framework, emphasizing how political factors affect environmental issues and decision-making (37, 38).

These pathways, not necessarily mutually exclusive, synergy and act in conjunction with other urban processes, forming specific mechanism in a particular context.

### 1.2. Summary of the past systematic review

International empirical research in this field has grown gradually in recent years, and it is increasing in popularity. Limited reviews are available discussing the relationship between health and gentrification, most of which reveal inconsistencies in existing research, with the direction and extent of impact varying according to context, definition of exposure, measurement factors, control variables, analytical methods and health outcomes, indicating the complexity of the conclusion. Some scholars have discussed gentrification in conjunction with other urban processes, for example Mehdipanah summarized the relationship between urban regeneration, gentrification and health, but the focus of his study does not involve a discussion of the above research design elements (39). Schnake-Mahl discussed the three neighborhood socioeconomic ascent processes of gentrification, urban development and urban regeneration in United States, but without elaborating on the definition of gentrification, health outcomes and mechanism (40). Santos provided an overview on the impact of neighborhood socioeconomic processes on older people (41). In systematic reviews with gentrification as a central theme, Jelks discussed green gentrification, focusing on health outcomes associated with green spaces and the pathways of impact, but did not discuss in detail the theoretical frameworks, definitions of exposure, measurement indicators, control variables, research methods and their influence on outcomes (42). Smith (43) and Bhavsar (44) discussed the association between gentrification measurements and health outcomes in American cities, but Smith did not explore the pathways and mechanism. Tulier (45) addressed the definition of gentrification, the mechanism and the temporal-spatial dimensions.

These reviews focused either on single type of gentrification, such as green gentrification (42), or on specific sub-populations (41), or on single geographical area such as the US or North America (43, 45). There is a lack of systematic summaries of health outcomes and a lack of comprehensive discussions that relates theoretical frameworks, definitions and measures of gentrification, analytical methods and influential pathways to health outcomes. Only Tulier (45) articulated a research framework linking definitions, mechanism and health outcomes, but only covered 17 papers. Other studies also examined limited amount of literature due to strict inclusion criteria—Smith (43) selected only 6 papers, Schnake-Mahl (40) selected 22 papers, Jelks (42) selected 15 papers and Cole (39) selected 29 papers, which affects the comprehensive discussion of the topic to some extent. This study covers more literature and focuses on a comprehensive discussion linking research design elements such as theoretical frameworks, definitions and measurement, analytical methods and influential pathways to health outcomes.

## 2. Methods

### 2.1. Study design

The literature comes from medical, social, geographical and environmental field, and approaches of data extraction and quality appraisal criteria vary from one another. In order to comprehensively and systematically mine the subject content, we have made some adjustments to construct our own data extraction and analysis procedures based on the guidelines of PRISMA 2020 and SCIE (46).

Our research question is: “How does gentrification process affect residents' health?”, which is structured using the PICOS approach to identify the components of the studies:

P: No explicit restrictions-entire population of a given area or specific population such as elderly people or teenagers;

I: gentrification;

C: neighborhood processes and dynamics other than gentrification;

O: health outcomes including direct outcomes and indirect outcomes such as health behavior/resources with high impact on health outcomes and well beings;

S: cross-sectional study design/longitudinal study design/phenomenological study design.

### 2.2. Inclusion criteria

**Figure 1:**
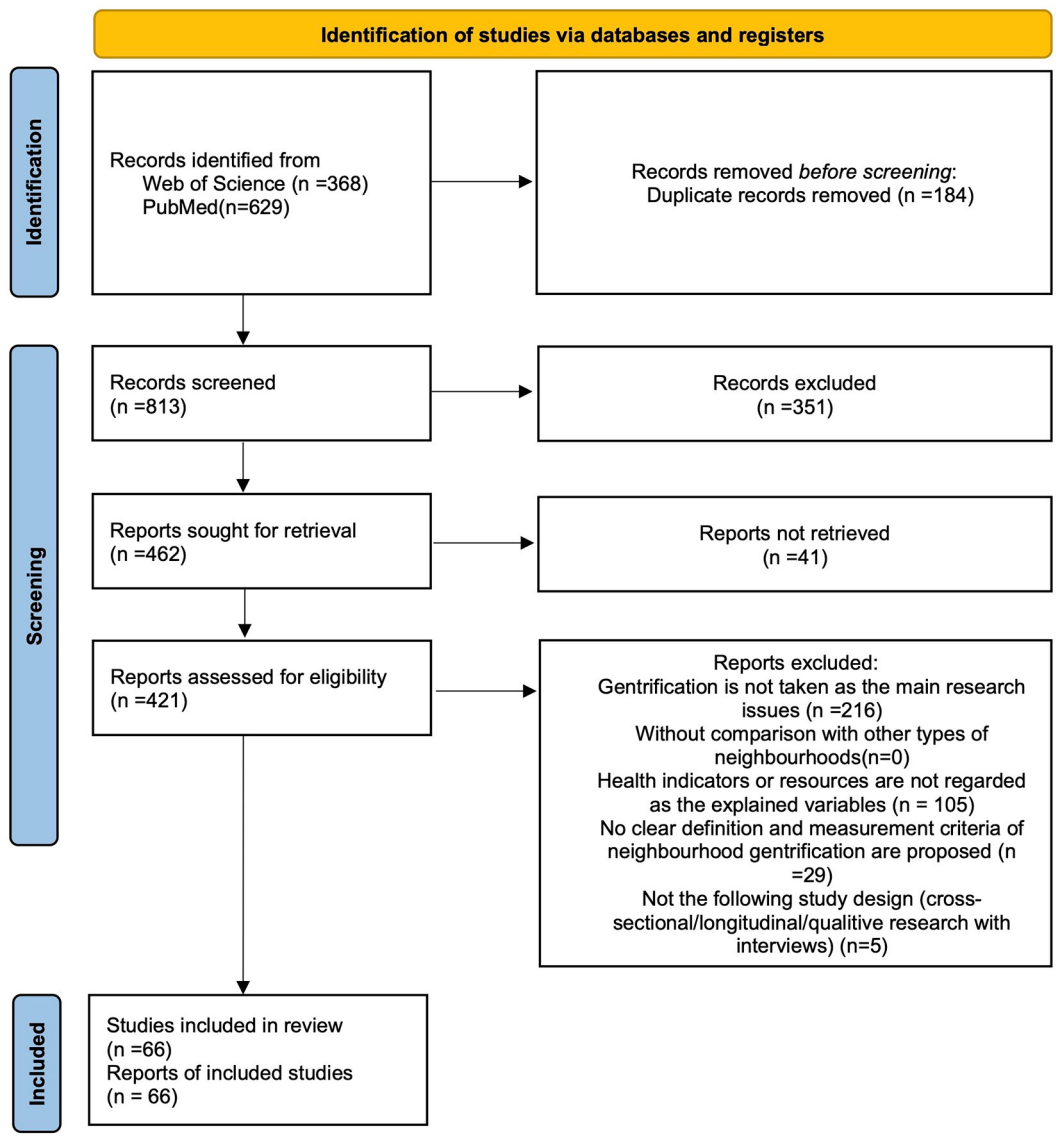
Flow chart following PRISMA guidelines.

First, the databases of Web of Science and PubMed were selected. Papers published in English in peer-reviewed journals and conference proceedings before January 1, 2023 are the main literature focused in this review. A range of search terms related to health and gentrification were proposed and the following combinations were used: “health” or “well-being”, “gentrification” or “neighborhood change” or “displacement” or “social upgrading” or “SES upgrading”. A total number of 66 papers were included after excluding items with duplicate content, no full text, or content irrelevant to the topic. As we mainly focus on empirical research with clear research methodology, so literature reviews or meta-analyses were also excluded. Furthermore, studies that did not taken the relationship between gentrification and health as the primary focus were excluded for the same reason. Studies examining the relationship between gentrification and health were considered to be eligible if they met the following criteria: (1). Neighborhood change is taken as the main research issue, and gentrification is regarded as one of the typical processes; (2). Various health indicators or resources are regarded as the explained variables; (3). Clear definition and measurement criteria of neighborhood gentrification are proposed, although this will vary by research (Figure 1). Disagreements were discussed to reach a consensus between 3 contributors in the above steps.

### 2.3. Data extraction and analysis

Data of those studies were extracted by reviewers independently and divided into 16 dimensions which have been tested before their use: author, article title, source title, publication year, abstract, research design, research methodology, focusing regions/countries/areas/cities, research scale (geographical), sample, data source, analytical model, index of gentrification, health outcomes, effects and key findings. The health outcomes are further clustered into broader categories according to the patterns of indicators. Qualitative and quantitative studies were combined to develop the analysis in terms of basic literature information, theoretical framework, analytical methods, definition and measurement of gentrification, health outcomes, and pathways.

Based on the standards of the SCIE guidelines (46), the quality evaluation of quantitative research through bias avoidance assessment was carried out among the screened studies. Since qualitative research and quantitative research are applicable to different assessment criteria, it is difficult to make comparison between them. Although qualitative research are more advantages to infer casual mechanism based on the specific case studies, but it is also rarely used to make general conclusions for the lack of subjective bias avoidance measurements. In addition, quantitative methodology is mostly used in the discussion of the relationship between gentrification and health outcomes in selected studies. Based on the above two reasons, quality assessment was exclusive to quantitative research, but in other parts of the review, the two were considered comprehensively. The evaluation items and scoring system for quality assessment is illustrated in Table 1, where the total score for a given study is equal to the sum of the score of individual items divided by the total score of the applicable items, resulting in a score between 0 and 1. Additionally, qualitative and quantitative studies were combined to develop the analysis in terms of basic literature information, theoretical framework, analytical methods, definition and measurement of gentrification, health outcomes, and pathways.

**Table 1:** Items and values of quality assessment.

**Bias assessment items**	**Values assigned**
**1. Measurements of gentrification**	
Are discussions of multiple factors included?	Yes (1), No (0)
Are Neighborhoods prejudged as gentrifiable areas?	Yes (1), No (0)
Is the extent of gentrification taken into consideration?	Yes (1), No (0)
Is the type of gentrification taken into account?	Yes (1), No (0)
**2. Settings of control groups**	
Comparison based on cohort analysis	2
Comparison based on cross-sectional analysis	1
No control group	0
**3. Solutions to endogeneity**	
Whether at least one of the following strategies are properly used or the problem of endogeneity is carefully discussed? research design (quasi-experimental research design), variables selection (instrumental variables), analysis models (difference-in-differences models, fixed effects model, GMM model, Heckman model and so on).	Yes (1), No (0)
**4. Spatiotemporal characteristics**	
Is spatial heterogeneity taken into account?	Yes (0.5), No (0)
Is temporal heterogeneity taken into account?	Yes (0.5), No (0)
**5. Robustness test**	
Has robustness test been performed?	Yes (1), No (0)
**6. Control variables**	
Are individual-level variables included?	Yes (1), No (0)
Are neighborhood-level sociospatial variables included?	Yes (1), No (0)
Are variables in respect of built environment included?	Yes (1), No (0)

## 3. Results

### 3.1. Basic information

**Figure 2:**
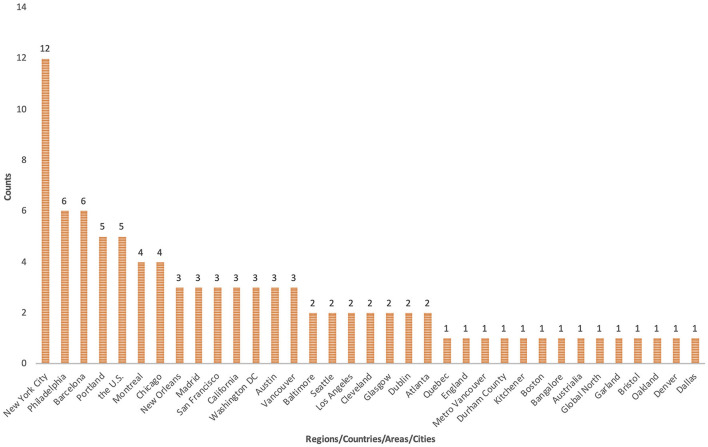
The regions/countries/areas/cities where the research objects located.

Based on the analysis of the documents, it can be seen that research on the relationship between gentrification and health has only begun to emerge in recent years, especially since 2017, at which time the output increased sharply. Research in this field is featured for its strong interdisciplinary nature, covering areas such as public environmental occupational health, geography, environmental ecology, urban studies, sociology, and public management. In terms of the geographical characteristics of the articles' authors, among the 14 countries involved, the United States, Canada, and Spain have an absolute advantage in the output of publications (91%), and 82 authors are from the United States (67%), which is somewhat associated with the severe racial issues in the United States. Figure 2 reveals the counts of the regions/countries/areas/cities where the research objects are located in 66 empirical publications. The current research mainly focuses on large cities in Europe and America that have undergone extensive gentrification. The North American cities, primarily represented by New York, have become the main focus, and the European cities that have received the most attention are Barcelona and Madrid.

### 3.2. Research design of the literature

Most of the literature in this field is based on ecological research designs, including cross-sectional research and longitudinal research: 28 papers (42%) were cross-sectional studies, nine (14%) were longitudinal studies, three (5%) used both cross-sectional and longitudinal research designs and 20 (30%) were phenomenological study design. To further clarify the causal relationship and eliminate interference factors, six papers (9%) adopted a quasi-experimental research design. These studies often used natural screening processes, such as public policy or natural disasters, to form qualified samples, and added before–after comparisons. For example, Lee (47) examined the Northridge earthquake in Los Angeles in 1994, and Schnake-Mahl (48) explored changes following Hurricane Katrina in 2005 to provide a reasonable basis for the randomness of neighborhood relocation into gentrifying and non-gentrifying neighborhoods. Overall, however, the conclusions drawn using quasi-experimental research designs with a low risk of bias do not appear to be fundamentally different from those drawn from longitudinal research designs. At the methodological level, 40 papers (61%) used quantitative methods, 20 papers (30%) used qualitative methods, and six papers (9%) used mixed methods. Mixed methods are usually based on the observation–analysis–interview procedure, with the quantitative approach used to initially determine the direction and degree of impact, and the qualitative analyses used to further discuss the mechanism of impact on health outcomes. In terms of the studies' conclusions, the impact of gentrification exposure on health outcomes identified in quantitative studies has no fixed direction, while the qualitative studies all point to a negative impact of gentrification.

**Figure 3:**
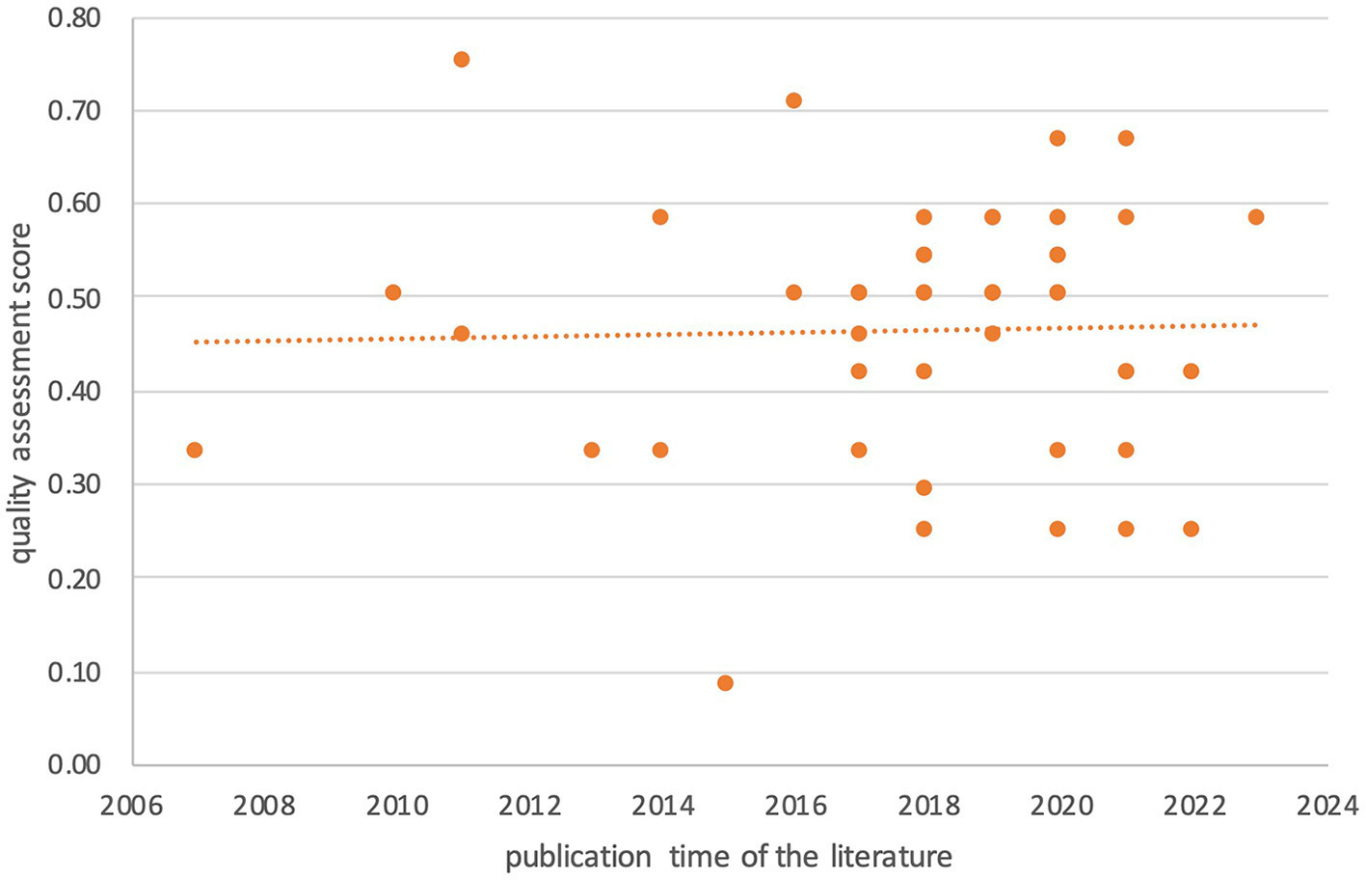
Bias avoidance score for quality assessment of quantitative studies.

The quality assessment scores of the 46 quantitative studies among 66 selected varies widely (scores ranged from 0.08 to 0.75). It is further evident from Figure 3 that only four (9%) of the studies have a low risk of bias (values >0.67), 78% have a moderate risk of bias (values between 0.33 and 0.67), and 13% have a high risk of bias (values lower than 0.33). The main factors that contribute to the bias avoidance score include the resolution of endogeneity and the consideration of gentrification spatiotemporal effects. 79% of the studies did not adopt any strategy to address endogeneity and 74% of the studies did not address spatial or temporal effects—only five studies considered spatial effects and only six studies considered temporal effects. In addition, 44% of the studies did not test for robustness. Built environment factors were also rarely included (2%). Compared to earlier studies, the average quality of the newly published literature is slightly higher.

### 3.3. Theoretical framework

The theoretical framework will determine the hypotheses being tested, and thus affects the choice of research methods. Most of the research on the relationship between gentrification and health can be included in the social epidemiology field, focusing on health inequality from microscopic perspective (30, 31, 48). Such studies can be broadly defined within the three theoretical frameworks mention in Section 1: Four (6%) papers can be classified into residential/exclusionary displacement theoretical framework, 12 (18%) adopted psychosocial theoretical framework, 14 (21%) adopted social production of disease/political economy of health framework, of which most of the research on environmental gentrification can be classified into this framework. Among the literature mentioned above, 4 simultaneously tested multiple theoretical frameworks and 2 explored the combined effects of gentrification and racial discrimination mechanisms.

Having a clear theoretical framework plays an important role in proposing research hypotheses and screening factors for consideration. But in general, due to the strong interdisciplinary nature of this field, most empirical research lacks a clear and complete theoretical framework. For example, most studies do not explain the subjects or agents that influence health outcomes (30).

### 3.4. Methods of analysis

The challenge of this type of research is to control for the interference of various factors and endogenous problems, such as sample selection bias. The analytical models used can be classified into the following three categories: regression model, survival analysis model and clustering model, among which, most studies used regression model. In cross-sectional studies, most researchers used multilevel regression models to assess the influencing factors at the individual and neighborhood levels, mapped individual attributes to family or community units, and compared gentrifying neighborhoods with other types of neighborhoods, including the differences between groups and those within groups. Individual studies use multiple regression models based on the characteristics of the data. For example, Izenberg (49) used California Health Interview Survey data to combine multiple regression models and Taylor series linearization methods instead of multilevel regression. The main reason is that the data were collected using random-digit dialing, and census tracts were not weighted differently. In longitudinal studies, in addition to multilevel models, two studies also used fixed-effects models to compare differences in health outcomes for individuals who moved or remained in different types of neighborhoods over time, controlling for covariates that did not change over time (gender and ethnicity). Goldenberg (50) further used the generalized estimating equation to measure the impacts of gentrification on multiple health-related mediator variables at the same time. Survival analysis model was used in one longitudinal study of diabetes incidence in Madrid and proved to be of low bias risk (51). In qualitative research, the method of thematic analysis is often used to identify and analyze major themes within original data such as transcripts of interviews with neighborhood stakeholders (52). Using integrative inductive-deductive approach to develop and refine a codebook including a list of codes and sub-codes relating to the research question, transcripts were coded and compared with the codebook, and quotations were selected to illustrate key themes of the research. These are based on the discussions and consultations of the research team, and both outcomes and mechanism can be derived from the analysis of these themes (53).

The common covariates included in the literature can be divided into two levels: individual and neighborhood. Individual-level covariates mainly include gender, ethnicity, age, socioeconomic status (income, education, assets, work status, and so forth.), house ownership, insurance status, marital status, medical history, and so on; neighborhood-level indicators mainly include ethnicity composition, age structure, proportion of immigrant population, poverty level, housing age, and others. Some studies also involve indicators at the family level, such as family structure and family income.

### 3.5. Definition and measurement of gentrification

First used to describe the displacement of working-class residents within a neighborhood by the wealthier middle class (54), gentrification has spread across the global into diverse urban contexts. At the same time, the definition and understanding has evolved to include more complex social and environmental changes, including the improvement of the neighborhood's physical environment, the increase of housing prices and rents, and the direct or indirect displacement of low-socioeconomic-status classes with high-socioeconomic-status classes. It may also be accompanied by new housing development projects, the invasion of high-end business, changes in neighborhood social networks and cultural characteristics, and so on. Defined by Davidson and Lees (23), the main components of gentrification are commonly mentioned as (1) reinvestment of capital; (2) social upgrading by incoming high-income groups; (3) changes of urban landscape; and (4) direct or indirect displacement of low-income groups. The definition of the gentrification process will affect the interpretation of the research conclusions and the guidance of policy making. In existing research, gentrification can be viewed as a passive or active process. Most studies regard gentrification as a process in which people with high socioeconomic status displace those with low socioeconomic status at the neighborhood level. For example, some studies used the changes in the proportions of different ethnic groups to distinguish the types of gentrification and reveal the differences in its impact on the health of different ethnic groups (51, 55, 56). In addition, some studies emphasized the residential displacement caused by gentrification. These articles confirmed that the health of displaced residents has been negatively affected (48, 57). From the perspective of political economy, gentrification is also considered as an urban strategy which facilitates the new opportunities for accumulation of capital (3). For example, some articles focused on gentrification caused by the eco-city policies (brownfield renewal and green infrastructure construction, and others.) or health policies (community sports facility renewal and green food initiatives, and others.), revealing that these policies attract the middle class to move in, reshape the reputation of the community, and further crowd out the life-support services for the disadvantaged, thus exacerbating community health inequality (58–60).

Existing studies used a variety of indicators to measure gentrification exposure, and no consensus has emerged on the appropriate number, value, and synthesis method of these indicators. In addition, there are also open questions about whether the measurement of gentrification exposure should reflect geographical differences (61). In quantitative research, there are usually two levels of criteria. The indicators are mainly derived from the measurement of changes of the socioeconomic status. Baseline indicators are used to determine that gentrification is eligible in the corresponding area: the level of the neighborhood unit (such as census unit, community group) is usually lower than the corresponding value (such as the median) of the larger administrative jurisdiction (such as city, county). The commonly used indicator in research is median household income; for areas where gentrification is eligible, the variation in the indicator over time is used to determine whether or not the corresponding area has experienced gentrification within a specific period of time (often consistent with the census cycle, such as 10 years). The variation must be higher than that in the wider jurisdiction over the specified time period. Most studies used one to six indicators, with the most commonly used factors including change in median household income, change in rent, and change in the share of bachelor's degree. In addition, some studies described gentrification as a staged process, using quintiles of standard scores to identify neighborhoods in mild, moderate, and advanced gentrification stages. Some studies also included indicators relating to investment and urban landscape to reflect the types and drivers of gentrification, such as gentrification with demographic shifts, private investment and state intervention (62). In addition to objective indicators, there are also studies using questionnaires to judge neighborhood changes and measure residents' subjective perceptions of gentrification (63). In studies employing qualitative methods, researchers typically define gentrification in terms of self-determined changes in population, rent, or household income, based on available information about the research area.

Differences in measurement indicators will lead to differences in research results. For example, Mujahid (64) compared three typical models: the Freeman model, Landis model, and UDP (Urban Displacement Project) model. The results indicated that the Freeman and Landis models identified the vast majority of census tracts in a given city as stable, with only 5.2 and 6.1% of the tracts, respectively, having gentrification characteristics. The UDP model identified 46.7% of the neighborhoods as at risk, undergoing, or experiencing advanced stages of gentrification and displacement.

“Neighborhoods” of different spatial scales are taken as the basic research units, which are mainly based on the differences in the data structures used. Most of these studies take the census tract as the research unit.^1^ Some scholars have pointed out that this kind of spatial scale is more suitable for research in the field of health (65), but others have adopted alternative spatial scales. One study on crime used ZIP codes (66), while two studies used PUMA (Public Use Microdata Area) units (57, 67),^2^ one on Portland used neighborhood boundaries (68), and two used neighborhood clusters (62, 67, 69).^3^ In terms of the research scope, most of the quantitative research focused on a certain city, and a few compared multiple cities or countries. Qualitative research mostly took one or several neighborhood units as the research scope. In terms of temporal scale, the time span of gentrification exposure is also mostly consistent with the census data, typically 10 years. In terms of conclusions, using residents' subjective perceptions to measure gentrification is more likely to lead to conclusions about the negative impacts on health, more consistent results. This trend has also been confirmed in related research on neighborhood effects (70).

### 3.6. Effects of gentrification on residents' health

The impact of gentrification on health is a complex process. Empirical research is revealing more detailed conclusions, varying due to differences in types of health outcomes, group characteristics, and time span characteristics. In the present studies, 41 papers (67%) revealed that the effect of gentrification on health occurs in a single direction, of which 31 noted a negative impact; 16 papers (26%) revealed a compound effect in multiple directions, showing positive/negative/neutral impacts.

#### 3.6.1. Differences in health outcomes

**Figure 4:**
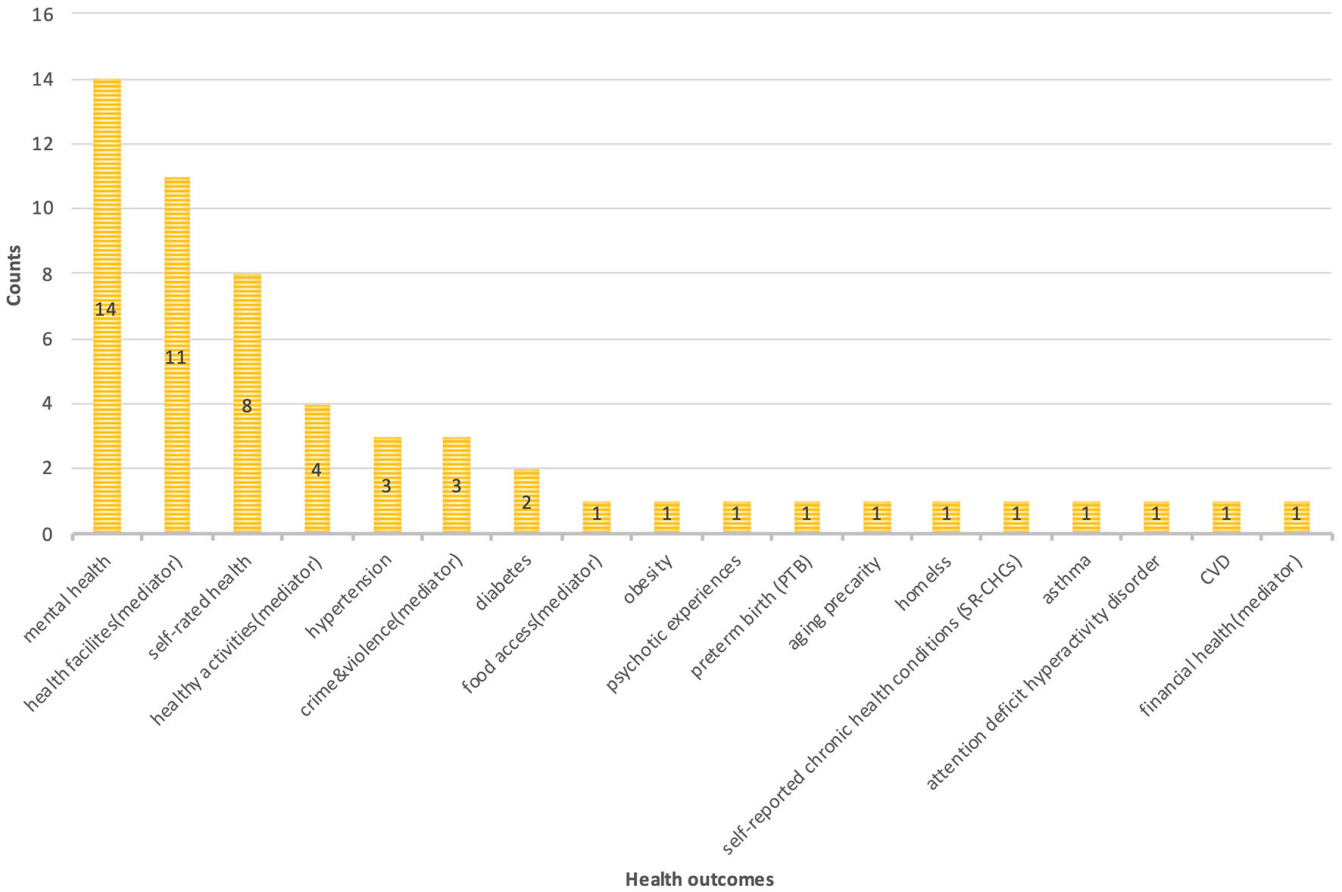
Types of health outcomes and publication counts in selected studies.

Figure 4 illustrates the types of health outcomes and research frequency characteristics that are found in the literature. The direct health outcomes mainly include mental health (psychological stress and anxiety), self-rated health status, chronic diseases (high blood pressure and obesity), premature birth, and others. Indirect health outcomes include mediators that can affect health, such as individual health behaviors (alcohol abuse, physical activity, visits to health care facilities, visits to green spaces and sports facilities, etc.), individual key health resources (credit score), neighborhood environmental risk factors (air pollution and crime rate), and neighborhood health resources (outdoor environmental quality, accessibility to green facilities and sports facilities, healthy food environment).

Mental health is most frequently mentioned in direct health outcomes. Most studies pointed out that, compared with non-gentrified neighborhoods, the environment of gentrified neighborhoods had a negative impact on the mental health of residents. This negative effect is not only more significant in low-income groups and long-term adults tenants (71), but also among the older adults and children (71, 72). An earlier qualitative study also confirmed the negative impact of gentrified neighborhoods on people with a history of mental illness (73). The difference in community ethnic composition also has a moderating effect. For example, gentrification characterized by an increase in the proportion of the white population (hereafter referred to as white gentrification) is more likely to create psychological stress and anxiety (55). In majority-Black gentrified neighborhoods, the mental health of residents is significantly worse than in neighborhoods mainly composed of other races (48). But a handful of studies have reached inconsistent conclusions, and these studies vary in their measures of gentrification and disease. For example, Narita (74) measured gentrification through subjective perception, and did not detect a significant correlation between gentrification and mental illness. However, Lim (57) used the frequency of visits to psychological clinics as an indicator of the mental health of residents in gentrified neighborhoods, and found no significant correlation between gentrification and visit frequency.

Self-rated health reflects the individual's subjective evaluation of their own health status. Most studies have shown that gentrification has a negligible impact on adults' self-rated health, but a negative impact on black people (48, 51), which is different from that of other ethnic groups, such as Hispanics and Asians (75). White gentrification was only associated with average or poor self-rated health among minorities, whereas both white and black gentrification were associated with average or poor self-rated health among black people, which may be related to the mental stress brought about by long-term racial discrimination and cultural displacement (51). However, a study of 500 cities in the United States reached the opposite conclusion: gentrification was positively and significantly correlated with residents' self-rated physiological health (76). The discrepancies in the results may be mainly due to the fact that the latter used neighborhood and city levels instead of individual and neighborhood levels in the measurement scale. Although compared with other studies, the coverage of different types of cities enhances the generalizability of the study, but there is also a risk of ecological fallacy.

Compared with other types of neighborhood environments, most studies have not found that gentrifying neighborhoods are positively correlated with the risk of chronic diseases, such as diabetes, hypertension, obesity, psychotic disorders, and asthma. Currently, there is only one study with 331 respondents, revealing that in gentrified neighborhoods, the self-rated chronic disease risk of black people is higher than that of other ethnic groups (77). Compared with gentrified neighborhoods, neighborhoods dominated by black people or poor neighborhoods have a higher probability of early detection of hypertension, and the prevalence of hypertension in gentrified neighborhoods is lower (69); however, there are no differences at the ethic level (78). Similar trends were observed for diabetes risk: compared with stable neighborhoods, residents in declining neighborhoods, newly built neighborhoods, and gentrified neighborhoods all had lower rates of diabetes (8, 9, and 11%, respectively) (79). In addition, gentrification has no significant effect on the risk of obesity (72), and the body mass index of whites who move into gentrified neighborhoods is significantly lower than that of whites who continue to live in low-income neighborhoods (48). This is possibly related to the fact that groups with high socioeconomic status are more likely to maintain good living habits.

Among the indirect health outcomes, health behaviors, including outdoor sports, in disadvantaged groups and ethnic minorities are negatively affected by gentrification due to discrimination, social exclusion, and privatization of public space (80). For short-term residents with a move-in period of < 5 years, gentrification is also positively correlated with alcohol abuse, which may become a stress-relieving strategy for the vulnerable group (75). In addition, some studies have pointed out that, since public drinking will be punished in gentrified neighborhoods, this may further lead to deeper social isolation and alcoholism (81).

The changes in social bonds and social identities brought about by the gentrification process will have positive or negative impacts depending on the group studied. For example, qualitative research on older adults revealed the loss of neighborhood social cohesion, social exclusion, and the breakdown of social bonds caused by gentrification (82, 83). Steinmetz-Wood (34) revealed the positive impact of gentrification on neighborhood collective efficacy through observation and interviews with 2,433 individuals; there were no significant differences in the perceptions of neighborhood collective efficacy between immigrants and native residents.

Most studies also proved that gentrification can reduce the health benefits for disadvantaged groups, thereby exacerbating health inequality, which reflects the moderating effect of gentrification. The more common form is environmental gentrification, accompanied by urban environment improvement measures to deal with environmental and ecological issues—brownfield renewal, air treatment, ecological transformation of old districts, and so on; it is gentrification induced by the redistribution of health environmental resources (84, 85). Relevant studies have revealed that the surrounding areas with better health environmental resources were often areas where gentrification occured intensively (86), and the ecological transformation of high-density central urban areas has further intensified the process (58, 59). This inequality is also reflected in the fact that the displacement induced by gentrification accelerates the migration of low-income groups to areas with higher levels of environmental risk factors, such as industrial areas with higher levels of exposure to harmful gases (87). While greater exposure to green space contributes to improved health outcomes, in gentrifying neighborhoods this is only true for higher-income groups with higher levels of education (88). The social isolation and exclusion brought about by gentrification make it difficult for groups with low socioeconomic status to actively participate in outdoor activities; they even regard green spaces as “disruptive green landscapes” rather than “healing green landscapes” (89). Research on the types of green spaces further revealed that only specific types of green facilities (green walks) had a significant positive effect on health (90). In addition to environmental gentrification, few studies have revealed the geographic and economic barriers gentrification poses to healthy food environments. A study revealed that food mirage^4^ often occured in gentrified areas where high-priced health food chains and grocery stores had increased, creating geographic barriers to affordable food for the underprivileged; meanwhile, affordable health food grocery stores were often forced to migrate to more distant areas (60). Economic barriers are reflected in the fact that gentrification forces residents to spend more of their income on housing, thereby reducing expenditures on food and exacerbating malnutrition among low-income groups (91).

#### 3.6.2. Group differences

In the above analysis, it can be seen that the impact of gentrification on the health of residents varies significantly across groups. In addition to the ethnic, income, educational, occupational differences in American cities, age, length of residence, migration characteristics, medical history, and house ownership also had a significant impact on the results. For example, a study on preterm birth in New York pointed out that, although the overall relationship between gentrification and preterm birth was not significant, compared with low-income neighborhoods, in highly gentrified neighborhoods, the probability of preterm birth in the Hispanic/Black group was higher, and that among non-Hispanic whites was lower (92). Different studies have revealed the indirect displacement caused by gentrification, which leads to the loss of social capital among the older adults (53, 82, 83, 93), often negatively affects mental health. A study found that low-income seniors in gentrified neighborhoods had higher levels of self-rated health, but high-income seniors had lower levels of mental health than their counterparts in poorer neighborhoods. Both low- and high-income seniors in gentrified neighborhoods had higher rates of depression and anxiety symptoms compared to their neighborhood peers (94). Furthermore, although gentrification has no significant impact on children's obesity and asthma, in rapidly gentrified neighborhoods, the probability of anxiety or depression among children from low-income families is significantly higher than in poor neighborhoods (72). A study of California revealed the differential effect depending on home ownership: for the long-term tenants, mental health was negatively correlated with gentrification, but no correlation between the two was observed in the newly settled group (71). Health risks of other minority vulnerable groups affected by gentrification were also revealed, such as the threat of housing security to transgender groups (95), geographical barriers to health testing services for sex workers and people who use drugs (50), increased displacement risk of people who inject drugs (66), among other factors.

#### 3.6.3. Time span differences

The results vary significantly due to the differences in exposure time and the heterogeneity of the gentrification process itself, which can be taken as a cumulative effect or a threshold effect. Based on a follow-up study in Los Angeles, Agbai (96) concluded that the longer the time lived in a gentrified neighborhood, the better the self-rated health status; in this case, there were no racial differences. Regarding mental health, a California-wide study also pointed out that gentrification had a negative impact on long-term residents (>5 years), while it had no significant impact on new residents (71). At the level of health behaviors, further research on California found that alcoholism was only significantly affected by gentrification in short-term immigrant groups (< 5 years) (75). Regarding the health benefits of green space, a mixed-methods study revealed that, for families with disadvantaged socioeconomic status, the frequency of visits to and satisfaction with green space decreased significantly within 3–5 years; that is, the long-term health benefits of community green space reduced over time, suggesting that the gentrification counteracts the long-term benefits of green space (97). In terms of financial health, the credit scores of residents in deeply gentrified neighborhoods increased, and the impact on long-term residents was greater than that on short-term residents; for residents displaced by gentrification, no matter what types of low-income groups were forced to move out of the neighborhoods, their credit scores would be negatively affected (98). At the same time, the impact of gentrification on financial health was also differential: deeply gentrified neighborhoods have a stronger impact on credit scores, while in early gentrified neighborhoods, this effect is not significant (98).

## 4. Pathways of gentrification's impact on residents' health

**Figure 5:**
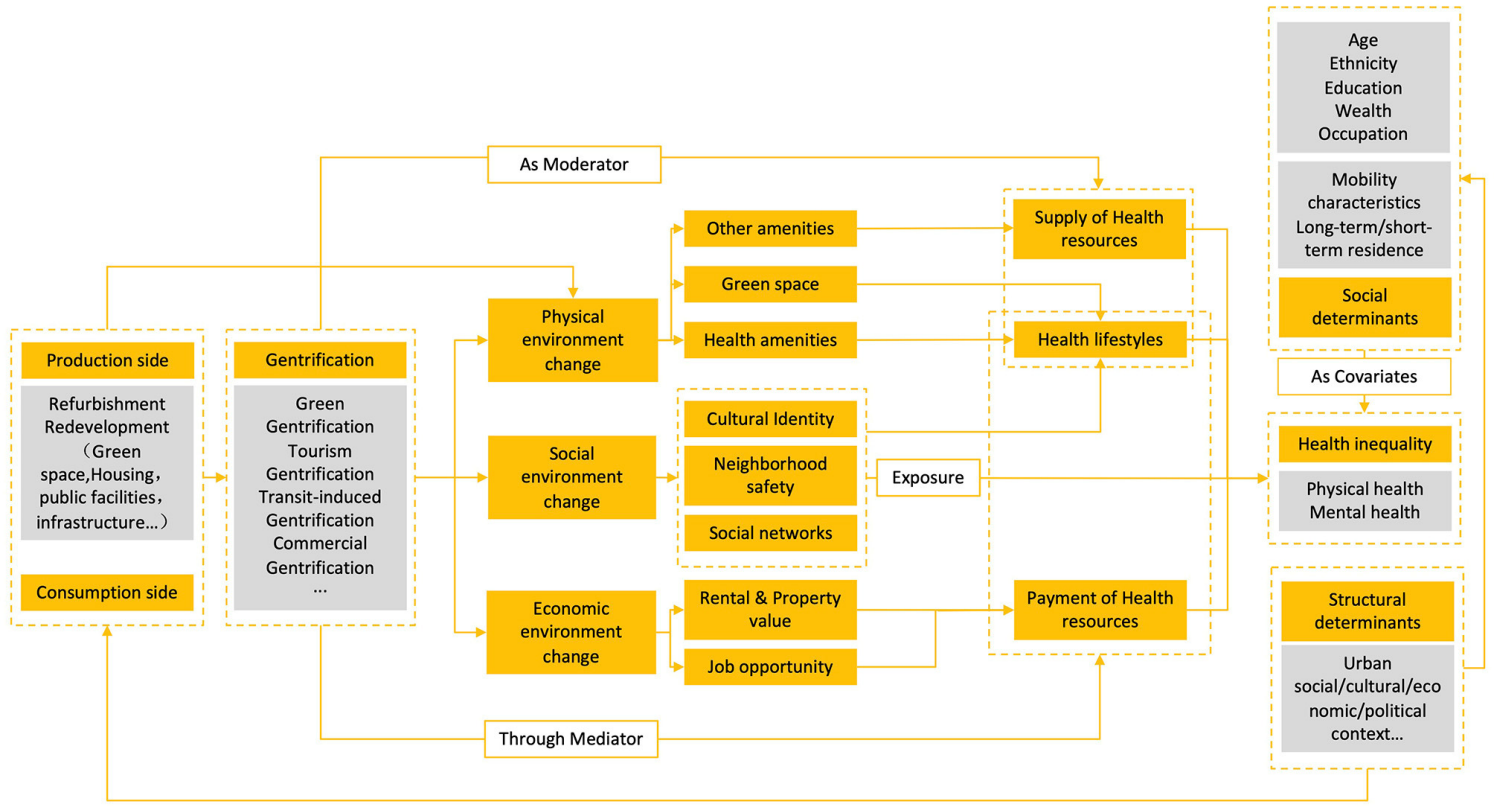
The pathways that illustrate the impact of gentrification on residents' health.

The validated pathways through which gentrification affects residents' health is summarized (Figure 5). These effects are mostly mediated through three levels: economic, social and physical environment change. However, these processes have relatively dissimilar pathways of impact on health, mainly as moderators, direct exposure and through mediators. Among them, the impact of changes in the physical environment acts mainly through moderating effects, in the sense that the gentrification process can alter how and to what extent population is affected. For instance, in gentrified neighborhoods, redevelopment aimed at improving residents' quality of life, such as brownfield restoration and building green spaces, can improve health benefits for high socioeconomic status groups, while having little effect on vulnerable groups, further exacerbating health inequalities (99). At the same time, these benefits are not evident in other types of neighborhoods compared to gentrified neighborhoods (99). Exposure pathway reveals the direct impact of gentrification process, primarily in the dimension of social environment change, such as the negative impact of socio-cultural displacement caused by gentrification on the mental health of disadvantaged groups. Furthermore, changes in the social and economic environment also have an impact through mediator factors, such as the ability to pay for health resources are undermined by increased housing and living costs, which are potential health risk factors for disadvantaged groups. In addition, structural determinants such as urban socio-cultural context, economic development stages and the political environment also determine distinctions in the groups affected, as well as the causes and types of gentrification. For instance, in many cities in the United States, the negative impact on minorities such as black people are repeatedly mentioned, while in Europe, the health problems of immigrant groups are more prominent. Gentrification, being restructured by urban process such as racial stratification, together with others, shape the landscape of health inequalities in a specific context. It is worth noting that while the above categorization is intended to be clear for discussion, the pathways are not necessarily mutually exclusive, that is, changes in the physical, social and economic environment may also interact to form more complex mechanism. For instance, improvements in the built environment may also increase the cost of living, resulting in an overlap between moderating and mediating effects; socio-cultural displacement may both directly affect mental health and potentially lead to unhealthy behaviors coping with stress.

First, the restructuring of the economic environment brought about by gentrification mainly acts on the individual dimension. Studies have confirmed that the process of gentrification, on the one hand, causing displacement pressure by increasing the living costs related to housing, thus depriving residents of key resources to deal with health problems, reducing stayers' ability to pay for essential services and goods to maintain health (98), and creating long-term stress (49, 100). On the other hand, the increase in living costs will also lead to direct residential displacement, forcing residents to move to areas with poor environment quality and a loss of access to adequate health protection resources (57). Regardless of the factors driving the gentrification process and the characteristics of its manifestation, research has revealed that mental health problems, such as chronic stress, anxiety, and depression, among disadvantaged groups, as well as older and younger groups, are caused by economic burdens (55, 90, 94). In addition, the incidence of other physical disorders, such as high blood pressure and fertility disorders including preterm birth, can also be seen as a direct result of the long-term effects of risk factors such as housing instability and financial pressures (69, 78, 92). This negative effect is further exacerbated by unhealthy behaviors in response to economic stress, such as alcohol and drug abuse (49).

Second, the reconstruction of the social environment mainly affects the neighborhood dimension. The changes in social capital caused by community instability not only affect the mental health of residents directly, but also lead to an uneven distribution of community health resources, and even cause conflicts and safety problems. Gentrification leads to changes in the neighborhood's demographic structure, altering social relations and community culture, which may cause social-cultural displacement for some vulnerable groups, increase relative deprivation, deepen social isolation, and affect residents' mental health (51, 101). It may also further induce unhealthy living habits to relieve stress. The social exclusion or social conflict that may be brought about by the mixing of different classes is a risk factor that contributes to crime (47, 60, 102, 103), causing a series of chain reactions, such as increased management costs and the less accessibility of public space, which makes vulnerable groups reduce their access to neighborhood health resources, such as green spaces (104, 105). For example, the intrusion of tourists brought about by tourism gentrification will cause social conflicts between residents and tourists (97). From another perspective, a higher level of social capital also enhance the stability of the neighborhood. For example, studies have confirmed that neighborhoods with high collective efficacy are less negatively affected by gentrification (25, 34).

Finally, the restructuring of the physical environment brought about by or inducing gentrification acts on both the individual and the neighborhood dimension. Besides the imbalanced distribution of health benefits caused by green gentrification, commercial displacement is also commonly mentioned, which is manifested as the reconfiguration of service amenities and commercial facilities in the community, transforming to business districts that cater to the tastes of the elite, resulting in the elevation and commercialization of basic health care facilities in the neighborhood. Residents with high socioeconomic status can benefit from this, but disadvantaged groups will experience reduced access to available public resources and health resources (101). For example, under the influence of tourism gentrification, the intrusion of shopping space catering to tourists has changed the commercial landscape of neighborhoods, hindered residents from obtaining sufficient affordable living necessities. Food mirage, namely, the influx of high-priced green food chain stores that cater to the tastes of the middle class but not affordable to disadvantaged groups, leading to the closure of traditional grocery stores and markets, poses similar effects (60, 106, 107).

## 5. Discussion

As been demonstrated in previous systematic reviews, gentrification serves as a potential contributor to health inequalities, but the results varies according to the measure of exposure, the methodology, the scale of the study, the context and the type of health outcomes (40, 41, 43–45). Through the review of relevant literature, the author believes that the current research can be improved in the following aspects:

First, as an interdisciplinary field, one of the challenges is that the concept of gentrification is difficult to define precisely and needs to be both generic and site-specific (43–45). Most of the literature used only a general socioeconomic perspective conceptualization of gentrification—that is, as a process of neighborhood-level socioeconomic upgrading, but other dynamics that came along and the root causes were more vaguely articulated. Only three articles revealed gentrification as an urban strategy of spatial restructuring; through this unequal process, spatial right is occupied by the higher socioeconomic classes. For example, Anguelovski (105) pointed out that the initiative of green food in supermarkets had led to the gentrification of food, and the original affordable grocery stores had been transformed to offer products that were not needed by vulnerable groups (Locally Unwanted Land Uses). The protests against this trend as an outcome of the process of gentrification, was only one example of the microcosm of the many conflicts and exclusions generated through the manipulation of discourse about food health and sustainability (105). Relatively speaking, research in the field of urban crime paid more attention to the specific definition of gentrification. For example, one study used three gentrification indicators to reflect different types of gentrification; it revealed that the observed crime rate increase was only related to government-led new-build gentrification (60).

Second, the conclusions drawn by existing studies are not consistent, and some are even contradictory, partly due to the inconsistency in the gentrification measurement methods, which is another challenge accompanied by the definition debate (42–45). For example, in quantitative research, the selection of the number of indicators, the selection of measurement standards, and decisions about how to synthesize them are somewhat subjective. Therefore, in future research, more site-specific measurement indicators should be determined for different research questions (108). Possible deviations in measurement methods should be clarified. For example, compared with the objective index measurement studies using subjective perception is more likely to draw negative conclusions about gentrification's impacts. In addition, different measurement methods should be compared, indicators that are site-specific for the macro-socioeconomic characteristics of different regions can be selected, and composite models can be used instead of isolated indicators to make the results more robust.

At the level of spatial scale, the MAUP (Modifiable Areal Unit Problem) (109) and spatial non-stationary or “within-area homogeneity” issue (110) should be considered. As for the MAUP, which means the results based on spatial data vary by the selected geographical sale, most studies focused on one city as the research scope and the census tract as the basic research unit. Whilst census tracts are commonly used as proxies for the neighborhood due to the availability of data, it is also crucial to be aware that the scale may not be a true picture of the geographic boundaries within which neighborhood dynamics occurs or is perceived by residents (45). Combining field surveys and big data can produce a finer resolution that reflects more variables and more accurately distinguishes gentrification from other urban processes, providing more nuanced conclusions for health studies. For instance, Hedin (111) identified Swedish urban gentrification features at the 100mX100m grid scale based on the ASTRID comprehensive microdata set. As for the spatial non-stationary issue, the spatial spillover effect of gentrification is often overlooked—that is, the local spatial differentiation characteristics caused by the radiation outward of gentrified neighborhoods to surrounding areas. On the one hand, this effect will shape the probability of gentrification in the neighborhoods and, on the other hand, the drastic spatial reconstruction caused by gentrification may also affect the surrounding area. At the same time, the analysis methods used in existing studies also lacked the identification of spatial non-stationarity; for example, the geographically weighted regression technology was not used to analyze the spatial differences in the degree of influence. This may be due to the fact that such studies mainly focused on the field of public health and lacked attention to the spatial dimension of geography.

The health outcomes reflected by existing studies mainly include mental health, self-rated health, and the prevalence of chronic diseases represented by obesity and hypertension. Future research may use electronic health data to measure more types of disease conditions longitudinally to more comprehensively uncover the characteristics of gentrification that affect health outcomes. Moreover, existing studies lacked an in-depth discussion of biological mechanism, nor do they include an analysis of how health behaviors, food, and outdoor space affect health outcomes. Such analyses require cooperation with public health, clinical medicine, and other disciplines, which will help reveal the impact mechanisms more accurately. Under the framework of ecosocial and related multilevel dynamics, the complementarity and integration of biology and sociology, the independent and cross-influence of multilevel factors, temporal evolution, and cumulative effects, among others (35), become the concern. For example, individual socioeconomic status affects the incidence of multiple diseases through multiple risk factors (environmental or health behavior), or key resources against disease risk (112). In terms of time span, existing studies all used the cycle that overlapped with the gentrification exposure period to estimate health outcomes, ignoring the lagging properties of diseases. Reasonable assumptions should be made about the impact of gentrification exposure duration, the staged characteristics of gentrification itself, and the incubation period of health diseases. Those should be reflected in the research designs, especially for crime rates, mental health, high blood pressure, and other factors.

At the level of research design, most of the existing research adopted cross-sectional research designs, using the health outcome index of a certain time point; therefore, it is difficult to make effective causal inferences. Using longitudinal or quasi-experimental research design to capture changes in health outcomes can more accurately help to identify the impacts of gentrification; moreover, these approaches are in line with the characteristics of gentrification as a progress. As for analytical methods, models that facilitate the solution of endogeneity problems, for example, the difference-in-differences model which proves to be effective has been rarely used in selected studies.

Finally, theoretical frameworks should be addressed to link the definition and measurement of gentrification, patterns of health outcomes, methodology and pathways. Although some studies can be roughly incorporated into at least one of the three frameworks mentioned at the beginning, more than half of the literature did not mention it and lacked a clear theoretical framework (31–33) or a discussion of the research context to guide the formulation of research hypotheses, the definition of place-based gentrification, selection of measurement indicators, identification of critical health problems and vulnerable sub-populations, which hindered the explanation of gentrification-induced health inequalities and the targeted guidance of policy interventions. Theories and contexts are often intertwined to form more complex phenomena, such as prominent racial stratification, tourism development, public housing development, and tax policies in certain regions and cities, which may act together with gentrification to shape patterns of health inequality, induce specific types of gentrification, or alter the extent to which gentrification affects health (112). Although the outcomes are associated with the intersection of multi-levels transformation, such as psychological stress induced by economic and social environment evolution, changes in neighborhood safety affected by both social and built environment transformation, and the positive and negative effects of gentrification can be counteracted in different contexts, the influential pathway at each level is somewhat dissimilar. A separate discussion and then synthesis of these pathways would be more effective in clarifying the issue. In some cases, displacement is conceived as both a component and a consequence of gentrification, but the two can be explained in different theoretical frameworks.

For instance, a disproportionate increase in economic burden is the key to involuntary residential displacement although there are also socio-cultural contributors (39). In this case the effect is mediated through accessibility to health resources. In such a framework, it is crucial to first clarify the causal relationship between gentrification and direct displacement, and to explore whether the dynamics of socioeconomic upgrading is due to out-migration or infill in terms of measuring gentrification. In the case of health outcomes, an examination of migration patterns and changes in financial health could be focused to reflect the impact on accessibility to health resources. Social environmental change may lead to sociocultural displacement, the effects of which on mental health and health behaviors are well documented (55, 71–73). In such a framework, where gentrification may act either as a direct exposure or through mediators, there is a need to identify the psychosocial mechanism (42), and in terms of measurement, incorporating subjective perceptions of neighborhood change may be more helpful to obtain convincing outcomes, with psychological distress and health behaviors becoming key health issues compared to others. In terms of sub-populations, minorities, groups that spend more time interacting with the neighborhood - the older people and children – could be potentially vulnerable residents. Transformation in physical environment, which is often accompanied by processes such as urban redevelopment, can be both a trigger and a consequence of gentrification, and will affect people's health lifestyles and the distribution of health resources. This process of intentional gentrification through public and private investment needs to be clarified through the mechanism of social production of disease or political economy of health, by specifying the agency of gentrification, looking specifically at the progress of redevelopment projects, revealing the causal relationship between physical environmental change and gentrification, clarifying the moderating role of gentrification. It is also convincing to include indicators revealing built environment and amenities change depending on the pattern of redevelopment. Under guidance of theoretical frameworks mentioned above, the use of quantitative methods in itself is not sufficient to examine the potential mechanism for the underlying relationships, and mixed approach is more advantageous for a clearer understanding of the pathways through which macro context and neighborhood dynamics exert their effects, thus is essential for public policy formulation.

## 6. Limitations and conclusions

The systematic review mainly focused on five levels of discussion on the relationship between gentrification and health outcomes: theoretical framework-analytical methods-definition and measurement of gentrification-effects-influential pathways, and proposes the possible measures to improve existing research quality. Although a wider range of health-related factors were covered as much as possible, the review was limited given that it did not covering sufficient databases and search terms, and some literature may still be missed. Contemporary gentrification has spread geographically well beyond the developed countries of Europe and the United States, and has taken on very different characteristics in the context of emerging developing countries. However, the literature on gentrification and health remains dominated by North America, particularly the United States, followed by Europe, with essentially no studies from the global South, possibly due to the more recent beginning of gentrification research in these regions and the limited availability of data. This imbalance in the distribution of the literature leaves much room for the development of research. Moreover, only quality assessment for quantitative research were conducted, which affected the comprehensiveness of the review to a certain extent.

The phenomenon of gentrification as one typical process of changing neighborhood environment proves to be a non-negligible factor affecting health segregation. The purpose of such research is to reveal the impact of this key social factor and how it imposes the influences. There are certain contradictions in the conclusions of the existing literature, and at the same time, there is insufficient guidance on how to reduce the negative impact of gentrification on health. The following measurement may help with that: The definition and measurement of gentrification should be both generic and site-specific; The comparison of various measurement methods should be made to enhance the robustness of the results. Furthermore, more consideration should be given to the impact of spatial issues. As for health outcomes, it is suggested to expand the scope of the discussion of health outcomes and strengthen the biological explanation of the influencing mechanisms. It is also necessary to determine the research time points according to the characteristics of the incubation period of different diseases. As for research design, applying longitudinal research design is more likely to improve the reliability. At last, theoretical frameworks should be addressed to link the definition and measurement of gentrification, patterns of health outcomes, methodology and pathways.

## Data availability statement

The original contributions presented in the study are included in the article/Supplementary material, further inquiries can be directed to the corresponding author.

## Author contributions

SD contributed to conception and design of the study, organized the database, statistical analysis, and wrote the manuscript.

## Footnotes

^1^In the United States, this geographical unit contains about 1,200–8,000 people or 480–3,200 households; in England and Wales, it generally contains 1,500 people (lower-level, superoutput areas).

^2^The scope of public micro data areas in the United States is larger than that of the census area, and the population contained in it varies greatly. For example, in the 2014 American Community Survey, the median population involved in New York PUMA is about 150,000.

^3^Each neighborhood cluster unit usually includes the scope of two census tracts.

^4^Areas where food is available but at a higher price.
