# Supplementary material for: Urban health inequality in shifting environment: systematic review on the impact of gentrification on residents' health
Source: Front Public Health. 2023 Jul 20;11:1154515. doi: 10.3389/fpubh.2023.1154515 (PMC10399630; doi:10.3389/fpubh.2023.1154515)
Supplement: Supplementary file 1 [file Data_Sheet_1.docx]

Supplementary Material

Systematic Review on the Impact of Gentrification on Residents’ Health in European and American Cities

Sun Delong*

*** Correspondence:** Sun Delong: xslx2005@126.com

# Supplementary Data

|  | Authors | Article Title | Source Title | Publication Year |
| --- | --- | --- | --- | --- |
| 1 | Morenoff, Jeffrey D.; House, James S.; Hansen, Ben B.; Williams, David R.; Kaplan, George A.; Hunte, Haslyn E. | Understanding social disparities in hypertension prevalence, awareness, treatment, and control: The role of neighborhood context | SOCIAL SCIENCE & MEDICINE | 2007 |
| 2 | Whittle, Henry J.; Palar, Kartika; Hufstedler, Lee Lemus; Seligman, Hilary K.; Frongillo, Edward A.; Weiser, Sheri D. | Food insecurity, chronic illness, and gentrification in the San Francisco Bay Area: An example of structural violence in United States public policy | SOCIAL SCIENCE & MEDICINE | 2015 |
| 3 | Gibbons, Joseph; Barton, Michael S. | The Association of Minority Self-Rated Health with Black versus White Gentrification | JOURNAL OF URBAN HEALTH-BULLETIN OF THE NEW YORK ACADEMY OF MEDICINE | 2016 |
| 4 | Huynh, M.; Maroko, A. R. | Gentrification and Preterm Birth in New York City, 2008-2010 | JOURNAL OF URBAN HEALTH-BULLETIN OF THE NEW YORK ACADEMY OF MEDICINE | 2014 |
| 5 | Smith, Richard J.; Lehning, Amanda J.; Kim, Kyeongmo | Aging in Place in Gentrifying Neighborhoods: Implications for Physical and Mental Health | GERONTOLOGIST | 2018 |
| 6 | Shmool, Jessie L. C.; Yonas, Michael A.; Newman, Ogonnaya Dotson; Kubzansky, Laura D.; Joseph, Evelyn; Parks, Ana; Callaway, Charles; Chubb, Lauren G.; Shepard, Peggy; Clougherty, Jane E. | Identifying Perceived Neighborhood Stressors Across Diverse Communities in New York City | AMERICAN JOURNAL OF COMMUNITY PSYCHOLOGY | 2015 |
| 7 | Cole, Helen V. S.; Triguero-Mas, Margarita; Connolly, James J. T.; Anguelovski, Isabelle | Determining the health benefits of green space: Does gentrification matter? | HEALTH & PLACE | 2019 |
| 8 | Lim, Sungwoo; Chan, Pui Ying; Walters, Sarah; Culp, Gretchen; Huynh, Mary; Gould, L. Hannah | Impact of residential displacement on healthcare access and mental health among original residents of gentrifying neighborhoods in New York City | PLOS ONE | 2017 |
| 9 | Izenberg, Jacob M.; Mujahid, Mahasin S.; Yen, Irene H. | Health in changing neighborhoods: A study of the relationship between gentrification and self-rated health in the state of California | HEALTH & PLACE | 2018 |
| 10 | Steinmetz-Wood, Madeleine; Wasfi, Rania; Parker, George; Bornstein, Lisa; Caron, Jean; Kestens, Yan | Is gentrification all bad? Positive association between gentrification and individual's perceived neighborhood collective efficacy in Montreal, Canada | INTERNATIONAL JOURNAL OF HEALTH GEOGRAPHICS | 2017 |
| 11 | Smith, Chris M. | The Influence of Gentrification on Gang Homicides in Chicago Neighborhoods, 1994 to 2005 | CRIME & DELINQUENCY | 2014 |
| 12 | Abel, Troy D.; White, Jonah | Skewed Riskscapes and Gentrified Inequities: Environmental Exposure Disparities in Seattle, Washington | AMERICAN JOURNAL OF PUBLIC HEALTH | 2011 |
| 13 | Maantay, Juliana A.; Maroko, Andrew R. | Brownfields to Greenfields: Environmental Justice Versus Environmental Gentrification | INTERNATIONAL JOURNAL OF ENVIRONMENTAL RESEARCH AND PUBLIC HEALTH | 2018 |
| 14 | Gibbons, Joseph; Barton, Michael; Brault, Elizabeth | Evaluating gentrification's relation to neighborhood and city health | PLOS ONE | 2018 |
| 15 | Burley, Blair Alexandra | Green infrastructure and violence: Do new street trees mitigate violent crime? | HEALTH & PLACE | 2018 |
| 16 | Versey, H. Shellae; Murad, Serene; Willems, Paul; Sanni, Mubarak | Beyond Housing: Perceptions of Indirect Displacement, Displacement Risk, and Aging Precarity as Challenges to Aging in Place in Gentrifying Cities | INTERNATIONAL JOURNAL OF ENVIRONMENTAL RESEARCH AND PUBLIC HEALTH | 2019 |
| 17 | Gibbons, Joseph | Are gentrifying neighborhoods more stressful? A multilevel analysis of self-rated stress | SSM-POPULATION HEALTH | 2019 |
| 18 | Linton, Sabriya L.; Cooper, Hannah L. F.; Kelley, Mary E.; Karnes, Conny C.; Ross, Zev; Wolfe, Mary E.; Friedman, Samuel R.; Jarlais, Don Des; Semaan, Salaam; Tempalski, Barbara; Sionean, Catlainn; DiNenno, Elizabeth; Wejnert, Cyprian; Paz-Bailey, Gabriela | Cross-sectional association between ZIP code-level gentrification and homelessness among a large community-based sample of people who inject drugs in 19 US cities | BMJ OPEN | 2017 |
| 19 | Dragan, Kacie L.; Ellen, Ingrid Gould; Glied, Sherry A. | Gentrification And The Health Of Low-Income Children In New York City | HEALTH AFFAIRS | 2019 |
| 20 | Swerdlow, M | Chronicity, nervios and community care: a case study of Puerto Rican psychiatric patients in New York City. | Culture, medicine and psychiatry | 1992 |
| 21 | Gibbons, Joseph; Barton, Michael S.; Reling, Timothy T. | Do gentrifying neighbourhoods have less community? Evidence from Philadelphia | URBAN STUDIES | 2020 |
| 22 | Sheringham, Jessica; Asaria, Miqdad; Barratt, Helen; Raine, Rosalind; Cookson, Richard | Are some areas more equal than others? Socioeconomic inequality in potentially avoidable emergency hospital admissions within English local authority areas | JOURNAL OF HEALTH SERVICES RESEARCH & POLICY | 2017 |
| 23 | Glick, Jennifer L.; Lopez, Alex; Pollock, Miranda; Theall, Katherine P. | Housing Insecurity Seems to Almost Go Hand in Hand with Being Trans: Housing Stress among Transgender and Gender Non-conforming Individuals in New Orleans | JOURNAL OF URBAN HEALTH-BULLETIN OF THE NEW YORK ACADEMY OF MEDICINE | 2019 |
| 24 | Fong, Polly; Cruwys, Tegan; Haslam, Catherine; Haslam, S. Alexander | Neighbourhood identification buffers the effects of (de-)gentrification and personal socioeconomic position on mental health | HEALTH & PLACE | 2019 |
| 25 | Triguero-Mas, Margarita; Anguelovski, Isabelle; Garcia-Lamarca, Melissa; Arguelles, Lucia; Perez-del-Pulgar, Carmen; Shokry, Galia; Connolly, James J. T.; Cole, Helen V. S. | Natural outdoor environments' health effects in gentrifying neighborhoods: Disruptive green landscapes for underprivileged neighborhood residents | SOCIAL SCIENCE & MEDICINE | 2021 |
| 26 | Sanchez-Ledesma, Esther; Vasquez-Vera, Hugo; Sagarra, Natalia; Peralta, Andres; Porthe, Victoria; Diez, Elia | Perceived pathways between tourism gentrification and health: A participatory Photovoice study in the Gotic neighborhood in Barcelona | SOCIAL SCIENCE & MEDICINE | 2020 |
| 27 | Schnake-Mahl, Alina; Sommers, Benjamin D.; Subramanian, S., V; Waters, Mary C.; Arcaya, Mariana | Effects of gentrification on health status after Hurricane Katrina | HEALTH & PLACE | 2020 |
| 28 | Lubitow, Amy; Tompkins, Kyla; Feldman, Madeleine | Sustainable Cycling For All? Race and Gender-Based Bicycling Inequalities in Portland, Oregon | CITY & COMMUNITY | 2019 |
| 29 | Cole, Helen V. S.; Mehdipanah, Roshanak; Gullon, Pedro; Triguero-Mas, Margarita | Breaking Down and Building Up: Gentrification, Its drivers, and Urban Health Inequality | CURRENT ENVIRONMENTAL HEALTH REPORTS | 2021 |
| 30 | Narita, Zui; Knowles, Kandra; Fedina, Lisa; Oh, Hans; Stickley, Andrew; Kelleher, Ian; DeVylder, Jordan | Neighborhood change and psychotic experiences in a general population sample | SCHIZOPHRENIA RESEARCH | 2020 |
| 31 | Izenberg, Jacob M.; Mujahid, Mahasin S.; Yen, Irene H. | Gentrification and binge drinking in California neighborhoods: It matters how long you've lived there | DRUG AND ALCOHOL DEPENDENCE | 2018 |
| 32 | Linda Diem Tran; Rice, Thomas H.; Ong, Paul M.; Banerjee, Sudipto; Liou, Julia; Ponce, Ninez A. | Impact of gentrification on adult mental health | HEALTH SERVICES RESEARCH | 2020 |
| 33 | Holt, Sidney L.; del Rio-Gonzalez, Ana Maria; Massie, Jenne S.; Bowleg, Lisa | I Live in This Neighborhood Too, Though: the Psychosocial Effects of Gentrification on Low-Income Black Men Living in Washington, DC | JOURNAL OF RACIAL AND ETHNIC HEALTH DISPARITIES | 2021 |
| 34 | Proulx, Jeffrey; Croff, Raina; Hebert, Michelle; Oken, Barry | Results of a mindfulness intervention feasibility study among elder African American women: A qualitative analysis | COMPLEMENTARY THERAPIES IN MEDICINE | 2020 |
| 35 | Goldenberg, Shira M.; Amram, Ofer; Braschel, Melissa; Moreheart, Sarah; Shannon, Kate | Urban gentrification and declining access to HIV/STI, sexual health, and outreach services amongst women sex workers between 2010-2014: Results of a community-based longitudinal cohort | HEALTH & PLACE | 2020 |
| 36 | Bhavsar, Nrupen A.; Shepherd-Banigan, Megan; Phelan, Matthew; Goldstein, Benjamin A.; Lunyera, Joseph; Diamantidis, Clarissa J.; Maciejewski, Matthew L.; Boulware, Ebony | Impact of Gentrification on Cardiovascular Disease Surveillance Using Data from the Electronic Health Record | CIRCULATION | 2019 |
| 37 | Barajas, Jesus M.; Braun, Lindsay M. | Are cycling and walking good for all? Tracking differences in associations among active travel, socioeconomics, gentrification, and self-reported health | JOURNAL OF TRANSPORT & HEALTH | 2021 |
| 38 | Agbai, Chinyere O. | Shifting neighborhoods, shifting health: A longitudinal analysis of gentrification and health in Los Angeles County | SOCIAL SCIENCE RESEARCH | 2021 |
| 39 | Anguelovski, Isabelle; Cole, Helen V S; O'Neill, Ella; Baro, Francesc; Kotsila, Panagiota; Sekulova, Filka; Perez Del Pulgar, Carmen; Shokry, Galia; Garcia-Lamarca, Melissa; Arguelles, Lucia; Connolly, James Jt; Honey-Roses, Jordi; Lopez-Gay, Antonio; Fontan-Vela, Mario; Matheney, Austin; Oscilowicz, Emilia; Binet, Andrew; Triguero-Mas, Margarita | Gentrification pathways and their health impacts on historically marginalized residents in Europe and North America: Global qualitative evidence from 14 cities. | Health & place | 2021 |
| 40 | Zayas-Costa, Montserrat; Cole, Helen V. S.; Anguelovski, Isabelle; Connolly, James J. T.; Bartoll, Xavier; Triguero-Mas, Margarita | Mental Health Outcomes in Barcelona: The Interplay between Gentrification and Greenspace | INTERNATIONAL JOURNAL OF ENVIRONMENTAL RESEARCH AND PUBLIC HEALTH | 2021 |
| 41 | Ong, Vanessa; Skinner, Kelly; Minaker, Leia M. | Life stories of food agency, health, and resilience in a rapidly gentrifying urban centre: Building a multidimensional concept of food access | SOCIAL SCIENCE & MEDICINE | 2021 |
| 42 | Iyanda, Ayodeji Emmanuel; Lu, Yongmei | Perceived Impact of Gentrification on Health and Well-Being: Exploring Social Capital and Coping Strategies in Gentrifying Neighborhoods | PROFESSIONAL GEOGRAPHER | 2021 |
| 43 | Iyanda, Ayodeji Emmanuel; Lu, Yongmei | Gentrification is not improving my health': a mixed-method investigation of chronic health conditions in rapidly changing urban neighborhoods in Austin, Texas | JOURNAL OF HOUSING AND THE BUILT ENVIRONMENT | 2021 |
| 44 | Cole, Helen V. S.; Anguelovski, Isabelle; Connolly, James J. T.; Garcia-Lamarca, Melissa; Perez-del-Pulgar, Carmen; Shokry, Galia; Triguero-Mas, Margarita | Adapting the environmental risk transition theory for urban health inequities: An observational study examining complex environmental riskscapes in seven neighborhoods in Global North cities | SOCIAL SCIENCE & MEDICINE | 2021 |
| 45 | Kim, Seung Kyum; Wu, Longfeng | Do the characteristics of new green space contribute to gentrification? | URBAN STUDIES | 2021 |
| 46 | Smith, Genee S.; McCleary, Rachael R.; Thorpe, Roland J., Jr. | Racial Disparities in Hypertension Prevalence within US Gentrifying Neighborhoods | INTERNATIONAL JOURNAL OF ENVIRONMENTAL RESEARCH AND PUBLIC HEALTH | 2020 |
| 47 | Fedina, Lisa; Mushonga, Dawnsha R.; Bessaha, Melissa L.; Jun, Hyun-Jin; Narita, Zui; DeVylder, Jordan | Moderating Effects of Perceived Neighborhood Factors on Intimate Partner Violence, Psychological Distress, and Suicide Risk | JOURNAL OF INTERPERSONAL VIOLENCE | 2021 |
| 48 | Oscilowicz, Emilia; Honey-Roses, Jordi; Anguelovski, Isabelle; Triguero-Mas, Margarita; Cole, Helen | Young families and children in gentrifying neighbourhoods: how gentrification reshapes use and perception of green play spaces | LOCAL ENVIRONMENT | 2020 |
| 49 | Collins, Alexandra B.; Boyd, Jade; Mayer, Samara; Fowler, Al; Kennedy, Mary Clare; Bluthenthal, Ricky N.; Kerr, Thomas; McNeil, Ryan | Policing space in the overdose crisis: A rapid ethnographic study of the impact of law enforcement practices on the effectiveness of overdose prevention sites | INTERNATIONAL JOURNAL OF DRUG POLICY | 2019 |
| 50 | Harris, Brandon; Schmalz, Dorothy; Larson, Lincoln; Fernandez, Mariela; Griffin, Sarah | Contested Spaces: Intimate Segregation and Environmental Gentrification on Chicago's 606 Trail | CITY & COMMUNITY | 2020 |
| 51 | Bilal, Usama; Glass, Thomas A.; del Cura-Gonzalez, Isabel; Sanchez-Perruca, Luis; Celentano, David D.; Franco, Manuel | Neighborhood social and economic change and diabetes incidence: The HeartHealthyHoods study | HEALTH & PLACE | 2019 |
| 52 | Schroeder, Krista; Klusaritz, Heather; Dupuis, Roxanne; Bolick, Ansley; Graves, Amy; Lipman, Terri H.; Cannuscio, Carolyn | Reconciling opposing perceptions of access to physical activity in a gentrifying urban neighborhood | PUBLIC HEALTH NURSING | 2019 |
| 53 | Versey, H. Shellae | A tale of two Harlems: Gentrification, social capital, and implications for aging in place | SOCIAL SCIENCE & MEDICINE | 2018 |
| 54 | Anguelovski, Isabelle; Connolly, James J. T.; Masip, Laia; Pearsall, Hamil | Assessing green gentrification in historically disenfranchised neighborhoods: a longitudinal and spatial analysis of Barcelona | URBAN GEOGRAPHY | 2018 |
| 55 | Lyons, Tara; Krusi, Andrea; Pierre, Leslie; Small, Will; Shannon, Kate | The impact of construction and gentrification on an outdoor trans sex work environment: Violence, displacement and policing | SEXUALITIES | 2017 |
| 56 | Gullon, Pedro; Bilal, Usama; Cebrecos, Alba; Badland, Hannah M.; Galan, Inaki; Franco, Manuel | Intersection of neighborhood dynamics and socioeconomic status in small-area walkability: the Heart Healthy Hoods project | INTERNATIONAL JOURNAL OF HEALTH GEOGRAPHICS | 2017 |
| 57 | Derkzen, Marthe L.; Nagendra, Harini; Van Teeffelen, Astrid J. A.; Purushotham, Anusha; Verburg, Peter H. | Shifts in ecosystem services in deprived urban areas: understanding people's responses and consequences for well-being | ECOLOGY AND SOCIETY | 2017 |
| 58 | Ding, Lei; Hwang, Jackelyn | The Consequences of Gentrification: A Focus on Residents' Financial Health in Philadelphia | CITYSCAPE | 2016 |
| 59 | Breyer, Betsy; Voss-Andreae, Adriana | Food mirages: Geographic and economic barriers to healthful food access in Portland, Oregon | HEALTH & PLACE | 2013 |
| 60 | Burns, Victoria F; Lavoie, Jean-Pierre; Rose, Damaris | Revisiting the role of neighbourhood change in social exclusion and inclusion of older people. | Journal of aging research | 2012 |
| 61 | Brennan Rhodes-Bratton 1, Andrew Rundle 2, Gina S Lovasi 3, Julie Herbstman 4 | The Relationship between Childhood Obesity and Neighborhood Food Ecology Explored through the Context of Gentrification in New York City | PUBLIC HEALTH JOURNAL | 2018 |
| 62 | Derek A. Kreager, Christopher J. Lyons and Zachary R. Hays | Urban revitalization and Seattle crime, 1982- 2000 | SOCIAL PROBLEMS | 2011 |
| 63 | YAN Y. LEE | Gentrification and crime: identification using the 1994 Northridge earthquake in Los Angeles | JOURNAL OF URBAN AFFAIRS | 2010 |
| 64 | Melstrom RT, Mohammadi R | Residential Mobility, Brownfield Remediation, and Environmental Gentrification in Chicago | Land Economics | 2022 |
| 65 | Hwang J, Shrimali BP | Shared and crowded housing in the bay area: Where gentrification and the housing crisis meet COVID-19 | Housing Policy Debate | 2023 |
| 66 | Williams PC, Alhasan DM, Krafty R, Coutts C, Miles-Richardson S, Jackson CL | A mixed methods approach to understand greenspace redevelopment in relation to objectively-and subjectively-measured sleep health among Black adults in Southwest Atlanta | Health & Place | 2022 |
